# Validation of four reference genes for quantitative mRNA expression studies in a rat model of inflammatory injury

**DOI:** 10.1186/1744-8069-10-55

**Published:** 2014-09-04

**Authors:** Roxanne Y Walder, Anne-Sophie Wattiez, Stephanie R White, Blanca Marquez de Prado, Marta V Hamity, Donna L Hammond

**Affiliations:** Departments of Anesthesia, University of Iowa, Iowa City, IA 52242 USA; Pharmacology, University of Iowa, 451 Newton Road, Iowa City, IA 52242 USA

**Keywords:** Complete Freund’s adjuvant, Hprt1, Mapk6, Actb, B2m, Pain, Nociception, Rostral ventromedial medulla, Dorsal horn, Housekeeping genes

## Abstract

**Background:**

Real-time quantitative PCR (qPCR) is a technique frequently used to measure changes in mRNA expression. To ensure validity of experimental findings, it is important to normalize the qPCR data to reference genes that are stable and unaffected by the experimental treatment to correct for variability among samples. Unlike in some models of neuropathic pain, reference genes for models of inflammatory injury have not been validated. This study examined four candidate reference genes in an effort to identify and validate optimal genes for normalization of transcriptional changes occurring in the dorsal horn of the spinal cord and the rostral ventromedial medulla (RVM) following intraplantar injection of complete Freund’s adjuvant (CFA).

**Results:**

The expression of hypoxanthine phosphoribosyltransferase 1 (Hprt1), beta-actin (Actb), mitogen-activated protein kinase 6 (Mapk6), and beta-2-microglobulin (B2m) was quantified in the dorsal horn and RVM of rats four days or two weeks after intraplantar injection of CFA or saline. The range of expression levels among these four genes differed by as much as 16-fold within the dorsal horn and the RVM. All four of these reference genes were stably expressed in both tissues and did not differ between saline and CFA-treated animals. Analyses using the statistical algorithms in geNorm and NormFinder programs determined that Mapk6 was the most stable gene and recommended the combination of Mapk6 and Actb, or Mapk6 and Hprt1, in such experimental conditions.

**Conclusions:**

This study validated the four genes Hprt1, Actb, Mapk6 or B2m and showed that any one or combination of two of them are good reference genes for normalization of mRNA expression in qPCR experiments in the spinal cord and RVM in the CFA model of inflammatory injury.

## Introduction

A better understanding of the molecular changes that occur during the development and maintenance of pain after inflammatory injury is fundamentally important for the development of effective therapies. Peripheral inflammatory injury activates signaling cascades in the peripheral and central nervous systems as well as in the immune system. These cascades lead to tissue swelling and increased sensitivity to both noxious and non-noxious stimuli that can persist long after the resolution of inflammation [1–4]. The underlying events entail transcriptional or translational changes in numerous genes and proteins including those involved in neurotransmitter release, receptor function and trafficking, subcellular signaling pathways, and regulation of ion channel expression and activity [1–4]. Identifying the nature of these changes can provide insights into the mechanisms of inflammatory injury and may elucidate new approaches for treating inflammatory pain.

Changes in gene expression that occur with peripheral inflammatory injury can be detected and measured in a sensitive and specific way using quantitative real-time polymerase chain reaction (qPCR) assays. Quantitative PCR is a frequently used technique that can be easily adapted to measure mRNA levels for any target protein and is the method of choice for absolute or relative quantification of mRNA expression. However, critics of qPCR maintain that it is often inadequately standardized and frequently inconsistent [5]. Some technical challenges inherent to the technique include isolating high quality RNA, identifying an efficient reverse transcriptase (RT) enzyme to generate cDNA, designing efficient and specific primers to amplify the desired mRNAs, and normalizing results to adequately validated reference genes. In 2009, Bustin et al. published the MIQE Guidelines: Minimum Information for Publication of Quantitative Real-Time PCR Experiments in an effort to standardize the information needed to ensure the relevance, accuracy, correct interpretation, and repeatability of qPCR experiments [6].

An essential component of qPCR is normalization of the target mRNA to a reference gene of interest in the same sample to control for variability associated with template input (amount of starting material) as well as RT and qPCR efficiencies. The reference gene mRNA should be stably expressed, and its abundance should show a strong correlation with the total amount of mRNA present in the sample [6]. Control reference genes were initially termed “housekeeping genes” because the genes were historically chosen from cellular maintenance proteins that are ubiquitously expressed and whose mRNA was generally thought to have uniformly unchanging expression in different cells and under different conditions. More recent reports have documented that housekeeping gene expression can vary substantially (e.g. in tumor environments and other pathophysiological states) and that the choice of reference gene can significantly impact the conclusions of a study [7–9]. The development of statistical algorithms (e.g. geNorm, BestKeeper or NormFinder [10–12]) to help researchers determine the most stable reference genes and most appropriate combinations of reference genes has advanced the field.

Many studies of the molecular mechanisms that underlie the development and maintenance of pain after inflammatory injury have used qPCR to examine changes in gene expression in the peripheral and central nervous systems. Yet, to our knowledge, rigorous validation of appropriate reference genes has not been undertaken. This study quantified the expression of four potential reference genes, hypoxanthine phosphoribosyltransferase 1 (Hprt1), beta-actin (Actb), mitogen-activated protein kinase 6 (Mapk6), and beta-2-microglobulin (B2m), in the dorsal horn of the spinal cord and rostral ventromedial medulla (RVM) of the rat. These regions play important roles in the development and maintenance of nociception after peripheral inflammatory injury [1, 4, 13, 14].

## Results

### Levels of Hprt1, Actb, Mapk6, and B2m mRNA in the dorsal horn and RVM of rats with persistent inflammatory injury

**Figure 1:**
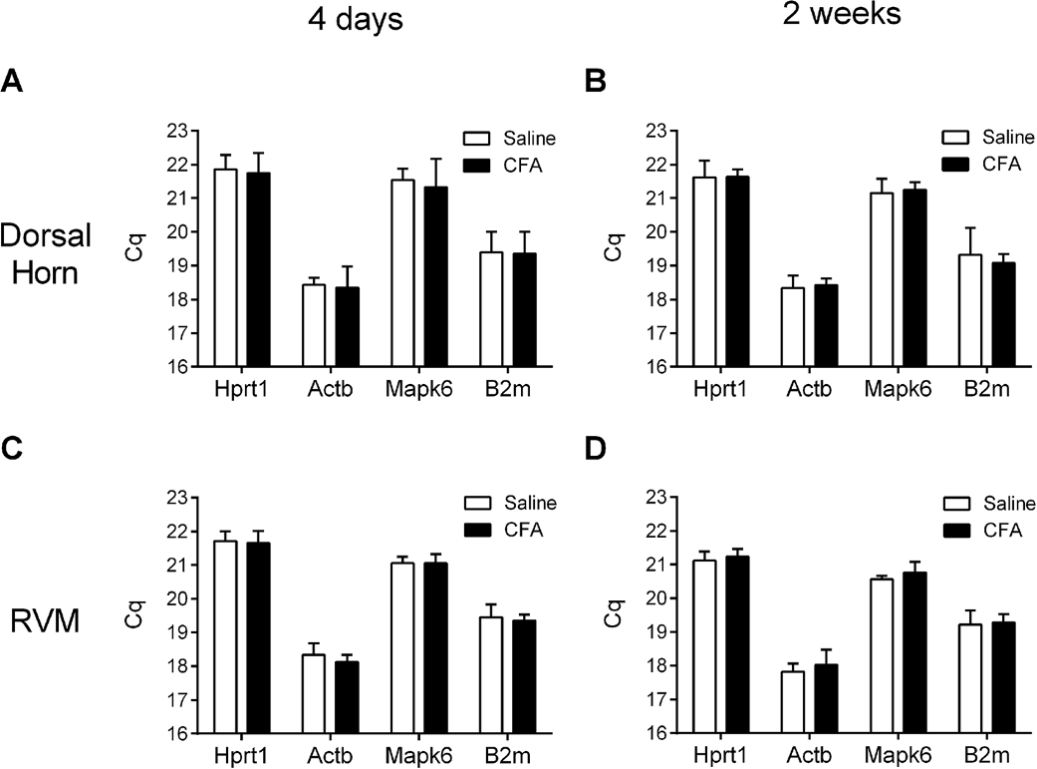
**Determination of transcript levels (Cq) of the candidate reference genes in the dorsal horn of the spinal cord (A, B) and RVM (C, D).** Levels were assessed four days **(A, C)** or two weeks **(B, D)** after intraplantar injection of CFA or saline. Values are the mean ± SEM of determinations made in 6–7 rats. In the dorsal horn, no differences were observed in the transcripts levels between saline- and CFA-treated rats, or between four days and two weeks, for any reference gene. In the RVM, transcript levels for each reference gene did not differ between saline- and CFA-treated rats. Although the Cq values at two weeks were statistically different from values at four days, the magnitude of the difference was about 0.2 Cq and unlikely to be biologically significant.

Transcripts for Hprt1, Actb, Mapk6, and B2m were readily detected in the dorsal horn of the spinal cord and the RVM of rats at four days (Figure 1A,C) and two weeks (Figure 1B,D) after intraplantar injection of saline or CFA. The four candidate reference genes were abundantly expressed in both tissues, with Cq values ranging from 21.11 to 21.85 for Hprt1, 17.81 to 18.42 for Actb, 20.56 to 21.54 for Mapk6, and 19.07 to 19.44 for B2m. The relative abundance of these mRNAs in both the dorsal horn and the RVM was Actb > B2m > Mapk6 > Hprt1 (P < 0.001).The levels of transcripts for all four candidate reference genes in CFA-treated rats were comparable to those in the corresponding saline-treated group in the dorsal horn (Figure 1A,B; P > 0.5 for all genes at both time points) and in the RVM (Figure 1C,D; P > 0.3 for all genes at both time points). The expression of each of the four reference genes in the dorsal horn at four days did not differ from that at two weeks in CFA- or saline-treated groups (P > 0.3, each gene). The expression of B2m in the RVM also did not differ at four days and two weeks in CFA- or saline-treated groups (P > 0.2). The expression levels of Hprt1, Actb, and MapK6 in the RVM at four days were statistically different from corresponding values at two weeks in saline- and CFA-treated rats (P < 0.05, each gene). However, the magnitude of the difference was less than 0.2 Cq and unlikely to be biologically significant.

### geNorm analysis of reference gene stability

**Figure 2:**
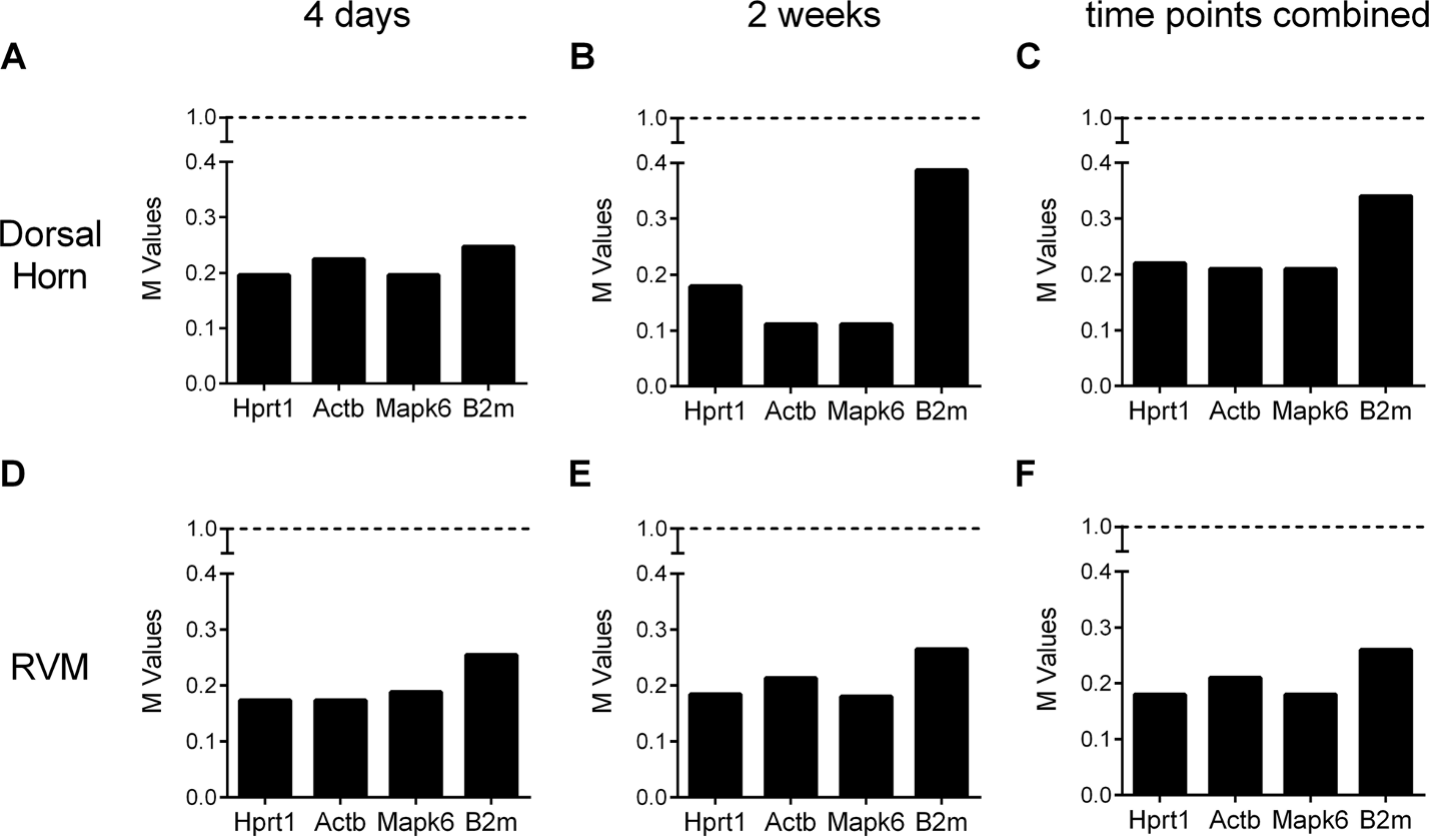
**Assessment of reference gene stability using the geNorm algorithm.** Average expression stability (M) for genes in the dorsal horn of the spinal cord **(A, B, C)** and in the RVM **(D, E, F)**, at four days **(A, D)** and two weeks **(B,E)** after intraplantar injection of CFA or saline, or for both time points combined **(C, F)**. All four genes were very stably expressed (M < 1.0) in both tissues and at all time points. The lowest M value indicates the most stable gene.

In the geNorm program, an M value under 1.0 is indicative of stable expression of mRNA [15]. In the dorsal horn, all four genes had M values less than 0.4, demonstrating that all four have high expression stability (Figure 2A,B). Comparisons of pair-wise variation in the stability values [12] of the four genes in the dorsal horn indicated that Mapk6 and Hprt1 were the most stable transcripts at four days and that Mapk6 and Actb were the most stable at two weeks. When the time points were pooled in the interest of following expression changes over time, geNorm analysis indicated that Actb and Mapk6 were the two optimal genes in the dorsal horn (Figure 2C). Similarly, in the RVM, all four genes exhibited high stability of expression at four days and two weeks after injury (Figure 2D,E). Comparisons of pair-wise variation in the stability value indicated that at four days Actb and Hprt1 transcripts were the most stable, whereas at two weeks Hprt1 and Mapk6 were the most stable (Figure 2D,E). Pooling data for both time points indicated that Hprt1 and Mapk6 were the two most stable genes in the RVM (Figure 2F).

### NormFinder analysis of reference gene stability

**Figure 3:**
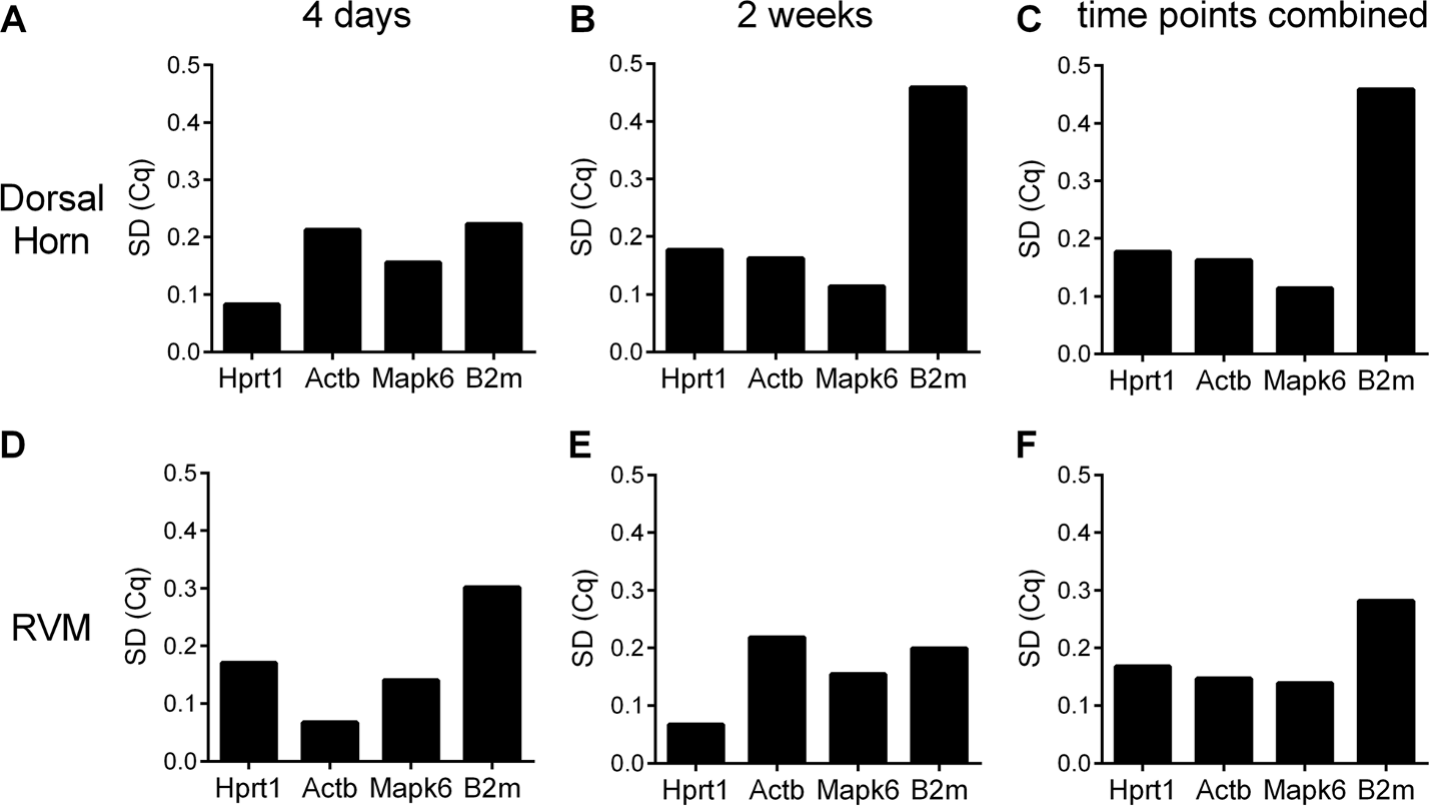
**Assessment of reference gene stability using the NormFinder analysis.** Stability values for genes in the dorsal horn **(A, B, C)** and in the RVM **(D, E, F)**, at four days **(A, D)** and two weeks **(B, E)** after intraplantar injection of CFA or saline, or for both time points combined **(C, F)** are shown. The lowest standard deviation (SD) indicates the most stable gene.

Expression stability for the four candidate reference genes was also calculated using NormFinder (Figure 3). In the dorsal horn, NormFinder analysis indicated that Hprt1 and Mapk6 were the best genes for normalization at four days and two weeks, respectively (Figure 3A,B). When the time points were pooled, NormFinder identified Mapk6 as the most stable gene for normalizing the expression data in the dorsal horn (Figure 3C). In the RVM, NormFinder analysis indicated that Actb and Hprt1 were the best genes for normalization at four days and two weeks, respectively (Figure 3D,E). When the data for both time points were pooled for analysis, NormFinder identified Mapk6 as the best gene for normalization in the RVM (Figure 3F). With respect to the best combination of two genes to normalize gene expression across time points, NormFinder identified Actb and Mapk6 in both the dorsal horn and RVM.

**Figure 4:**
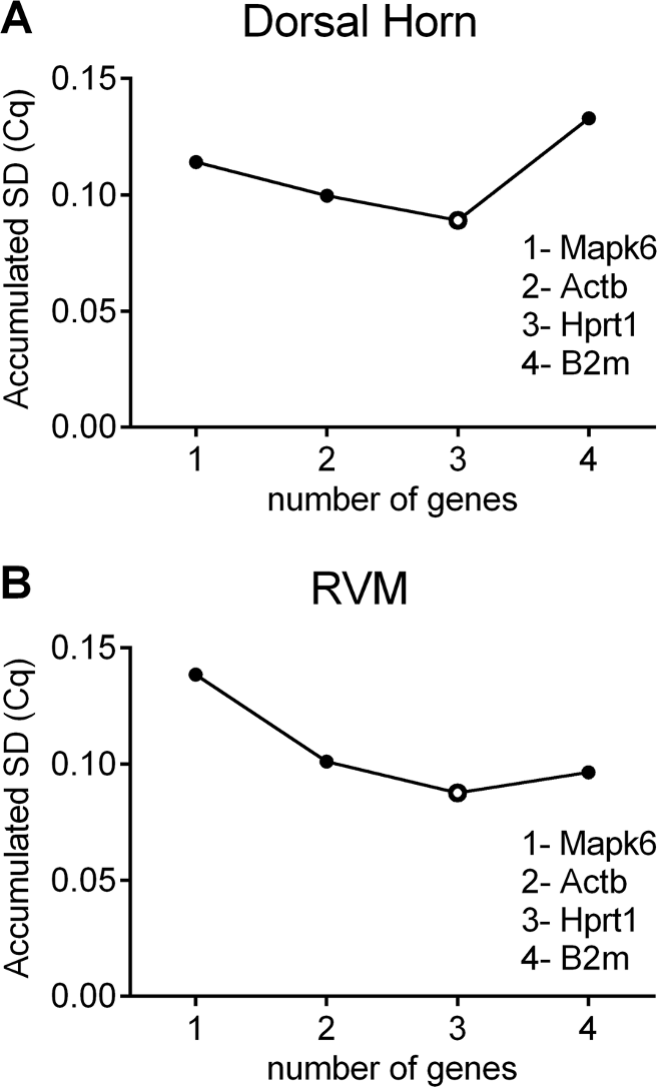
**Determination of the optimal number of reference genes by the NormFinder algorithm.** Stepwise calculation of stability began with the most stable gene (Mapk6), followed by serial addition of the next most stable gene and calculation of the accumulated standard deviation (circles). In both the dorsal horn **(A)** and in the RVM **(B)**, the optimal number of reference genes to be used in qPCR experiments was three (open circle).

NormFinder also includes an analysis of the optimal number of genes for normalization. In both dorsal horn and RVM, NormFinder identified the combination of Mapk6, Actb, and Hprt1 as optimal. However, the addition of one or two more genes caused at best a 0.06 decrease in the accumulated standard deviation, much less than the 0.15 that is considered a significant change (Figure 4). Thus, although the use of all three genes is predicted to be optimal, one gene would be acceptable for normalization of a target gene when resources are restricted.

## Discussion

As qPCR methodology has matured and its use has become more widespread, an increasing number of reports have demonstrated that the expression of classically used reference genes can vary substantially depending on the experimental conditions [7–9, 16]. Moreover, radically different conclusions can be reached depending on the reference gene used for normalization [16–19]. A PubMed literature search (March 20, 2014; search algorithm available from authors) identified 260 papers in English that described qPCR in rat models of nociception. The majority of the papers (83%, n = 216) normalized the expression of the target genes to a single reference gene. Glyceraldehyde 3-phosphate dehydrogenase (Gapdh) was used in 48% (n = 104), Actb in 25% (n = 54), Hprt1 in 4% (n = 8), and other housekeeping genes in the remaining 23% (n = 50) of the papers. Only 5% (n = 12) of the papers used more than one reference gene for normalization. When the search was restricted to studies using the CFA model of inflammatory injury in the rat, only 16 studies were identified. These 16 papers also normalized qPCR data with the same reference genes and in the same relative ratios as observed in the total 260 papers. To our knowledge, the present study is the first to specifically validate the use of reference genes for normalizing qPCR data in the CFA model of inflammatory injury.

Optimally, the transcript of the reference gene(s) selected for any given qPCR analysis will be expressed in an abundance comparable to that of the mRNA of interest [6]. This study identified four different reference genes – Mapk6, Actb, Hprt1, and B2m – that can be used for the normalization of qPCR data in the CFA model of inflammatory injury in rats. These four genes were selected because they are broadly expressed, involved in different homeostatic functions (Table 1) and are not co-regulated [12]. Moreover, two of these genes were also studied for validation as potential reference genes in the spared nerve injury [20] and spinal nerve ligation [21] models. The addition of our data allows the assessment of the stability of those genes over different pain models of chronic pain, possibly strengthening their utility as reference genes. The Cq values of the four genes ranged from 17 to 21.85 Cq, reflecting a 2^4^ or a 16-fold difference in expression. Each gene was stably expressed within each time point: four days (subacute injury) and two weeks (chronic injury) after injury in CFA-treated animals compared to corresponding saline-treated animals. Interestingly, the Cq values for these genes in the RVM were very similar to those in the dorsal horn. The NormFinder algorithm indicated that Mapk6 was the reference gene with the least variability and therefore likely the best reference gene of the four in our experimental conditions. It also indicated that the combined use of three reference genes (Mapk6, Actb and Hprt1) was optimal for normalization in both the dorsal horn and the RVM. However, the gain in stability between the use of a single gene and the use of three genes is minimal, as shown by the very small changes in the accumulated SD (< 0.06) between one and three genes, indicating that any one of these three genes would be acceptable as a unique reference gene.

**Table 1:** **Primer sequences for the candidate reference genes**

Gene name	Accession number	Function	Sequence	Product size (base pairs)
Hprt1	Genbank: NM_012583	Purine synthesis in salvage pathways	*Forward* 5′-CTCATGGACTGATTATGGACAGGAC *Reverse* 5′-GCAGGTCAGCAAAGAACTTATAGCC	123
Actb	Genbank: NM_031144	Cytoskeletal structural protein	*Forward* 5′-CCGCGAGTACAACCTTCTTG *Reverse* 5′-GCAGCGATATCGTCATCCAT	81
Mapk6	Genbank: NM_031622	Member of the Ser/Thr protein kinase superfamily	*Forward* 5′-TAAAGCCATTGACATGTGGG *Reverse* 5′-TCGTGCACAACAGGGATAGA	129
B2m	Genbank: NM_012512	Beta-chain of major histocompatibility complex class I molecules	*Forward* 5′-CGAGACCGATGTATATGCTTGC *Reverse* 5′-GTCCAGATGATTCAGAGCTCCA	114

The need to report qPCR data in a standardized format is now widely accepted, and recommendations for the uniformity and reproducibility of qPCR experiments are listed in the MIQE Guidelines [6]. These guidelines emphasize the need to control for sample-to-sample variation by normalization with reference genes. Normalization is necessary for reliable qPCR studies because the starting material, RNA extraction, RT efficiency, and qPCR efficiency can vary among experiments. Moreover, gene expression is highly tissue-specific and often varies based on the pathophysiological status of the organism or experimental treatment. Thus, it is imperative that a pilot study be conducted at the outset of any study to identify the optimal reference gene or combination of genes for that specific experiment. Unfortunately, very few validation studies for the use of reference genes exist in the pain field, and all are restricted to nerve injury models. Several studies have identified suitable qPCR reference genes in dorsal root ganglia in models of neuropathic pain [11, 21, 22]. For example, Mapk6 and Gapdh were identified as the two most stably expressed genes in an analysis of L4 and L5 dorsal root ganglia following L5 spinal nerve ligation [21]. Hprt1 and 18S were validated as stable genes for normalizing expression levels in the dorsal root ganglia in the spared nerve injury model [22, 23], although this conclusion was not shared by Piller et al. [20]. Seven commonly used reference genes, of which Actb was the most stable, were ranked and validated as good reference genes in spinal cord tissue in the spared nerve injury model of neuropathic pain [20]. These reports support the conclusion that the optimal reference gene(s) will be specific to each experimental condition, and likely gender as well.

Gapdh was the single most frequently used reference gene in the literature search but was not included in this analysis for several reasons. Over the last decade, the transcription of Gapdh has been reported to be significantly regulated in different experimental settings and is variable in different tissues [24–26]. Moreover, the rat genome contains 329 Gapdh pseudogenes [27, 28], some of which are transcribed and have the same sequence as the active Gapdh transcript and, as such, can be detected by primers and amplified. For example, amplification using a published Gapdh primer pair [29]: forward (5′-ACCACGAGAAATATGACAACTCCC) and reverse (5′-CCAAAGTTGTCATGGATGACC), designed to amplify a product of 100 base pairs, can give the same size amplicon from numerous transcripts. Performing an *in silico* PCR test by inserting the primer pair sequences for Gapdh in the UCSC genome website revealed 53 PCR products, each with 100 base pairs and 18 additional matches ranging from 75 to 120 base pairs, derived from genes on 16 different chromosomes. Other published primers for Gapdh yielded similar findings [20, 21]. Even if no genomic DNA contamination exists, and the RNA samples are treated with DNase, a more optimal qPCR assay design is one that does not amplify any pseudogenes. Similarly, Actb has pseudogenes [28], but the primers used in the present study were carefully selected to amplify Actb cDNA and not any of the pseudogene sequences. The UCSC *in silico* PCR search identified only one unique product in the rat genome with the Actb primers used here. Hprt1 also has pseudogenes, but the primers selected for this study are unique: one primer spans the splice junction of the gene, and when the primer pair is tested *in silico*, no genomic product was identified since the primers can only amplify the mRNA.

## Conclusions

In summary, this study identified four reference genes that are stably expressed in the dorsal horn and the RVM four days and two weeks after intraplantar injection of CFA or saline. Each assay for the four reference genes was designed with primers that were unique for their target and would not amplify pseudogenes. Mapk6 was the most stable reference gene, although all genes tested met the criteria for a validated reference gene. We suggest that using any one or combination of two of these assays (e.g. Mapk6 and Actb, or Mapk6 and Hprt1) for normalization of data would yield accurate and reproducible results when studying mRNA expression in the spinal cord and RVM in the CFA model of inflammatory injury.

## Methods

### Animals and inflammatory injury model

Male Sprague–Dawley rats (200–350 g; Charles River, Raleigh, NC, USA) were housed in pairs in the University of Iowa Animal Care Facility in rooms with a 12 hr light/dark cycle with water and food provided *ad libitum*. All studies were conducted in accordance with the Guide for Care and Use of Laboratory Animals published by the National Institutes of Health following the guidelines of the International Association for the Study of Pain. The experiments were approved by the University of Iowa Animal Care and Use Committee (protocol 1107156), and care was taken to minimize the number of animals used and their suffering.

Persistent inflammatory injury was induced by intraplantar injection of CFA. Adult rats were lightly anesthetized with isoflurane, and the thickness of the hindpaw was measured with digital calipers. The plantar surface of the left hindpaw was injected with 150 μl of CFA (150 μg of *Mycobacterium butyricum*, Calbiochem, La Jolla, CA, USA) or sterile-filtered saline at pH 7.4. The rats were returned to their cages and singly housed for four days or two weeks, depending on the experiment. After the rats were euthanized, the thickness of the hindpaw was measured to verify the presence of inflammation. Four days after injection of CFA, the thickness of the ipsilateral hindpaw had increased from 6.1 ± 0.1 to 9.4 ± 0.4 mm (N = 12; P < 0.01). The thickness of the hindpaw in the saline-treated cohort was unchanged (6.2 ± 0.1 mm; N = 13; P > 0.3). Two weeks after injection of CFA, the thickness of the ipsilateral hindpaw had increased from 6.0 ± 0.1 to 9.0 ± 0.3 mm (N = 10, P < 0.01) whereas the thickness of the hindpaw in the saline-treated cohort was unchanged (6.0 ± 0.1 mm, N = 10, P > 0.4). Measures of nociception were not made to minimize stimulation.

### qPCR – Quantitative Real-Time PCR Analysis

#### Tissue dissection

On the designated day, the rats were euthanized by CO_2_ inhalation and a 2-mm transverse slice of the brainstem containing the RVM was immediately isolated on ice and frozen on a platform on dry ice. To obtain the RVM, a 1.5-mm diameter tissue punch (Harris Unicore, Ted Pella Inc., Redding, CA), centered on the midline immediately above the pyramids, was removed from the frozen slice of brainstem tissue. The remainder of the slice was fixed in 10% formalin containing 30% sucrose to allow the verification of the site of the RVM tissue punch. The L4 and L5 portion of the spinal cord was removed, chilled on an ice-cold platform, and the ipsilateral dorsal horn was excised. The tissues were stored at -20°C in RNA*later*™ (Ambion, Life Technologies, Carlsbad, CA) until RNA isolation.

#### Primer design

DNA oligonucleotide primers were synthesized and purchased from Integrated DNA Technologies (Coralville, IA). The sequence of the forward and reverse primers for each of the four reference genes are listed in Table 1. The primers for Mapk6, B2m, Hprt1, and Actb are described elsewhere [21, 30, 31], and checked with the Primer 3 software (http://biotools.umassmed.edu) [32]. Each qPCR assay consists of primers that hybridize to sequences that lie on different exons or span a splice junction, separated by one or more introns, such that when qPCR is conducted, only the cDNA sequence is amplified. The Ensembl database (http://useast.ensembl.org/) was used to examine the genomic structure of the gene and its transcripts. Special care was used in the selection of primers for Actb and Hprt1, which are known to have pseudogenes [12, 27, 28]. Contaminating genomic DNA, if present, will not amplify any product under the reaction conditions. Thus, only the specific mRNA targets will be measured. When the forward and reverse primer sequences are entered into the UCSC genome bioinformatics website (http://genome.ucsc.edu/) and tested *in silico* against rat genomic DNA, only a single product is identified for Mapk6 and Actb primers. In the case of the Hprt1 and B2m primers, no genomic match is found because one of primers spans a splice junction and can only hybridize with the correct cDNA sequence. In addition, the PCR amplicons for each qPCR assay were cloned into pSC-A, the PCR cloning vector, according to the manufacturer’s protocol (StrataClone PCR Cloning Kit, Agilent Technologies, Santa Clara, CA), and sequenced at the Iowa Institute of Human Genetics, Genomics Division. Results were aligned with the Genbank sequence for the intended mRNA using the NCBI BLAST program to confirm the specificity of each primer pair. When the primers are used in a qPCR assay, they amplify a unique species with a sharp melting curve, and no primer-dimer products are detected.

#### ***RNA isolation, RT, and***qPCR

Total RNA was isolated from dorsal horn and RVM tissue according to the manufacturer’s protocol (RNeasy Lipid Tissue Mini Kit, Qiagen). Briefly, each tissue sample was homogenized in 1 ml of QIAzol lysis reagent. The lysate was extracted with chloroform, centrifuged, and the supernatant saved in a clean tube. The supernatant fraction was mixed with an equal volume of 70% ethanol and loaded on the column. Samples were treated with DNase I while on the column for 15 min at room temperature. After washing, the RNA was eluted from the column using RNase-free water. The concentration of total RNA was measured on a Nanodrop spectrophotometer (ND1000 3.8.1, Thermo Scientific, Wilmington, DE). The RNA integrity number was determined for 20% of the RNA samples as further RNA quality control and routinely showed values > 9.0 (out of 10) (Agilent Model 2100 Bioanalyzer, Santa Clara, CA). Reverse transcription was performed according to the manufacturer’s protocol using 100–600 ng of purified RNA and the SuperScript VILO cDNA synthesis kit (Life Technologies, Carlsbad, CA) in a 20 μl reaction volume. No-reverse transcriptase controls were also run for each RNA sample. The qPCR was performed with each well containing the cDNA product generated from 5 ng of input RNA, forward and reverse primers (12 nM), and the fluorogenic DNA-binding dye iQ™ SYBR® Green Supermix (Bio-Rad, Hercules, CA) in 20 μl. Reactions were performed in triplicate on a Bio-Rad CFX96 thermocycler (Bio-Rad, Hercules, CA). The cycle conditions were: 50°C for 2 min, 95°C for 10 min followed by 40 cycles of (95°C for 15 s, 60°C for 1 min and 72°C for 1 min), and then 95°C for 1 min and 55°C for 1 min. A thermal melting curve was generated from 55 to 95°C, at increments of 0.5°C for 10 s. No reverse transcriptase and no template controls for each primer pair were also tested in triplicate and did not amplify any product. Amplification efficiencies, calculated using the Bio-Rad CFX Manager 3.0 software, were similar for all primers and averaged at E = 101.2% ± 2.8, r^2^ ≥ 0.993 ± 0.002, slope = -3.30 ± 0.07. Cq values for each sample were calculated by the Bio-Rad CFX96 software (Bio-Rad, Hercules, CA).

### geNorm and NormFinder

These two programs are available in the GenEX 6 software package (http://genex.gene-quantification.info/). The geNorm program calculates gene expression stability (M) for each reference gene as the average of a pairwise variation for the reference gene relative to the others. The stepwise analysis allows for a ranking of the genes according to the calculated M value. The lower the M value, the more stable the reference gene.

The data for NormFinder are organized in groups: e.g. A) four day RVM, B) two week RVM, C) four day dorsal horn, and D) two week dorsal horn. The program calculates intra-group and inter-group variations in the expression of the reference genes from which a stability value is generated for each reference gene. The candidate gene with the lowest stability value is considered the most stable reference gene. By calculation of the standard deviation of the data, the NormFinder program selects the best number of reference genes for an experiment.

### Statistical analysis

Data are expressed as mean and S.E.M. The rank order of the expression of the four genes was analyzed by a one-way ANOVA followed by Bonferroni’s multiple comparison post-hoc test. A two-way ANOVA (factors: treatment and time) was used to compare the Cq values between saline- and CFA-treated rats, as well as between the four day and two week time points for each gene within each tissue. A P ≤ 0.05 was considered significant.

## Abbreviations

Actb: Beta-actin
B2m: Beta-2-microglobulin
CFA: Complete Freund’s adjuvant
Gapdh: Glyceraldehyde 3-phosphate dehydrogenase
Hprt1: Hypoxanthine phosphoribosyltransferase 1
Mapk6: Mitogen-activated protein kinase 6
qPCR: Real-time quantitative polymerase chain reaction
RT: Reverse transcriptase
RVM: Rostral ventromedial medulla.
